# Process of implementing and delivering the Prevention of Delirium system of care: a mixed method preliminary study

**DOI:** 10.1186/s12877-019-1374-x

**Published:** 2019-12-31

**Authors:** Mary Godfrey, John Green, Jane Smith, Francine Cheater, Sharon K. Inouye, Keith Hurst, John Young

**Affiliations:** 1 Academic Unit of Elderly Care and Rehabilitation, Bradford Teaching Hospitals NHS Foundation Trust, Bradford, West Yorkshire UK; 2 Leeds Institute of Health Sciences, University of Leeds, Leeds, West Yorkshire UK; 3 School of Health Sciences, University of East Anglia, Norwich, UK; 4 Beth Israel Deaconess Medical Center, Harvard Medical School, Boston, MA USA; 5 Aging Brain Center, Marcus Institute for Aging Research, Hebrew SeniorLife, Boston, MA USA; 6 Independent Researcher, Kings Lynn, Norfolk Island

**Keywords:** Delirium prevention, Older people, Hospital care, Hospital volunteers, Implementation

## Abstract

**Background:**

Delirium is a frequent complication of hospital admission among older people. Multicomponent interventions which can reduce incident delirium by ≈one-third are recommended by the National Institute of Health and Care Excellence. Currently, a standardised delirium prevention system of care suitable for adoption in the UK National Health Service does not exist. The Prevention of Delirium (POD) system of care is a theory informed, multicomponent intervention and systematic implementation process which includes a role for hospital volunteers. We report POD implementation and delivery processes in NHS hospital wards, as part of a feasibility study.

**Methods:**

A comparative case study design and participatory, multi-method evaluation was performed with sequential six month preparatory and six month delivery stages. Six wards in five hospitals in Northern England were recruited. Methods included: facilitated workshops; observation of POD preparatory activities; qualitative interviews with staff; collection of ward organisational and patient profiles; and structured observation of staff workload.

**Results:**

POD implementation and delivery was fully accomplished in four wards. On these wards, implementation strategies informed by Normalization Process Theory operated synergistically and cumulatively. An interactive staff training programme on delirium and practices that might prevent it among those at risk, facilitated purposeful POD engagement. Observation of practice juxtaposed to action on delirium preventive interventions created tension for change, legitimating new ways of organising work around it. Establishing systems, processes and documentation to make POD workable in the ward setting, enhanced staff ownership. ‘Negotiated experimentation’ to involve staff in creating, appraising and modifying systems and practices, helped integrate the POD care system in ward routines. Activating these change mechanisms required a particular form of leadership: pro-active ‘steer’, and senior ward ‘facilitator’ to extend ‘reach’ to the staff group. Organisational discontinuity (i.e. ward re-location and re-modelling) disrupted and extended POD implementation; staff shortages adversely affected staff capacity to invest in POD. Findings resulted in the development of ‘site readiness’ criteria without which implementation of this complex intervention was unlikely to occur.

**Conclusions:**

POD implementation and delivery is feasible in NHS wards, but a necessary context for success is ‘site readiness.’

## Background

Delirium is a frequent complication of hospital admission in older people [1]. It is associated with distress for patients, their families and staff [2, 3]; increased mortality, protracted lengths of hospital stay, functional and cognitive decline, and new long stay care admissions [4, 5]. Multicomponent delirium prevention interventions have been shown to reduce incident delirium in hospitalised patients by ≈one-third [1, 6, 7]. The National Institute of Health and Care Excellence (NICE) recommend implementation of these interventions in the National Health Service (NHS) in England and Wales [8]. However, no standardised delirium prevention system of care is currently available that is suitable for widespread NHS adoption.

In the first research phase we developed a novel delirium prevention intervention and multifaceted implementation strategy, the Prevention of Delirium system of care (POD) that would be sustainable in NHS hospital wards [9]. Our starting point was the evidence encapsulated in the NICE delirium guideline [8] and the Hospital Elder Life Program (HELP) [6, 10], a proven delirium prevention system of care with a dedicated HELP team to deliver it (Elder Life nurse specialist, Elder Life specialist volunteer co-ordinator and trained volunteers). We conducted empirical research on how delirium was understood and preventive action accomplished by NHS hospital ward staff, employing Normalisation Process Theory (NPT) [11, 12] as a sensitising lens to examine barriers to prevention and to inform implementation strategies. NPT was considered most appropriate given its focus on micro-social processes that affect implementation of new practices in an organisational setting. The evolving model of delirium prevention comprising ward cultural and practice change supported by volunteers was explored through participatory action research in three hospitals with multidisciplinary groups of clinical and care staff, voluntary services managers and volunteers, and patient and carer representatives, iteratively with review of the implementation literature [13–16]. In this study, part of the second research phase, we aimed to investigate the process of implementing and delivering POD in context of dynamic, acute hospital wards, and to inform changes to the intervention and its implementation in a feasibility trial.

### The prevention of delirium system of care (POD)

POD targets people at risk of delirium (≥65 years; pre-existing cognitive impairment; current fractured hip; severe illness). It comprises actions summarised in protocols addressing ten modifiable risk factors associated with the development of delirium among vulnerable patients. Action is directed at changing ward practice with patients to optimise hydration and nutrition, reduce environmental threats, increase orientation to time and place, improve communication, support and encourage mobility, and effect better pain, infection and medication management. Intervention content is based on individual assessed need, and its form is flexible, for example, ‘stimulating activities’ could include music, games or reminiscence work, delivered individually or in groups. Since these practices are pertinent to optimising patient experience and recovery, systematic engagement of wards in delirium prevention has potential to enhance patient care.

NPT constructs, *coherence, cognitive participation, collective action* and *reflexive monitoring* [11, 12], provided a sensitising framework to structure implementation design aimed at installing and embedding delirium prevention in care routines:

- Training staff on delirium and preventive practices to facilitate their purposeful engagement in POD, making it meaningful and worthwhile to invest in (*coherence*)*;*
- Structured observation of existing practice by members of the implementation team, and its juxtaposition with delirium preventive interventions to show how POD is different to ‘usual care’, legitimating work relating to it and building a community of practice around it (*cognitive participation*);
- Establishing systems, processes and associated documentation to make POD workable and integrated into ward routines to facilitate enactment of POD practices by staff and volunteers individually and collectively (*collective action*);
- Ongoing review of how the structures, systems and processes put in place and the practices reproduced deliver on valued outcomes (*reflexive monitoring*)*.*

It was envisaged that implementation and delivery would be led by hospital and ward staff with the authority and legitimacy to pursue the work (‘implementation team’). A manual was provided containing guidance, tools, training material and exemplar documents (posters, volunteer roles and tasks) for use, modification or replacement [9], and researchers offered ongoing support. The product of implementation planning was a bespoke delirium prevention system of care, the content of which was flexible and adapted to local contexts. Nevertheless, the principles underpinning POD, the steps in the change process to facilitate action, and the functions they performed, were standardised and generalisable [17].

## Methods

Between 2012 and 2014, we undertook a two-stage study, collecting qualitative and quantitative data prospectively during consecutive six-month periods: preparing implementation; and delivering POD. We used a case study approach [18, 19] and mixed methods to develop understanding about POD implementation and delivery processes in context of hospital wards that varied in their organisational features (ward model, staffing, and dependency) and patient profile.

### Setting and sample

Five NHS hospitals of varying size and locality type were purposively selected. Within hospitals, we purposively sampled six wards in which older people dominated. The multi-level strategy was to enable cross-case comparison of factors contributing to variation in implementation and delivery.

### Data collection

We developed patient profiles from routinely collected, anonymised data (sex, age on admission, length of hospital stay, discharge destination) for all ward admissions, during each study stage. Ward organisational features (physical environment, layout, staffing, skill mix, bed occupancy and patient dependency) drew on staff interviews and structured observation.

Researchers conducted ethnographic observation on each ward [total: 85 h]. This included: observation of preparatory implementation activities (volunteer training sessions, informal introductory events for volunteers and staff, and meetings in which staff discussed the audit/observations they had carried out and action plans flowing from them); informant conversations with implementation team members individually and together over time; collection of relevant preexisting documents (assessment, care and discharge planning documentation) and new materials developed and used during delivery. Researchers observed ward practices (staff/patient interaction, multi-disciplinary team meetings and handovers) to capture ‘usual care’ pertinent to delirium and how it changed with POD. Qualitative interviews with ward staff and volunteers were carried out toward the end of delivery to explore perceptions of POD implementation, delivery and impact [total: 28]. The research team maintained a diary /events log to provide a contemporaneous account of unfolding processes and action in time: communication with teams (emails, telephone conversations and face-to-face discussion), problems encountered, solutions arrived at, and relevant contextual features (e.g. changes to organisational priorities and procedures; staffing; ward models and profiles). We organised a one-day central workshop to share learning, build on what was similar and unique in different ward contexts and elicit participants’ perceptions of implementation.

A ‘dependency-acuity-quality’ method was adopted at the start of implementation planning and during delivery to gauge its impact on ward staff workload [20, 21]. Researchers conducted structured observations of staff activities linked to patients’ dependency/acuity during a 24 h period over six shifts (two early; two late; and two night shifts) at each time-point [total: equating to 2500 h staff time]. Activities included: direct care (face-to-face bedside care), indirect care (patient-related but not face-to-face activity and associated work (e.g. ‘hotel’- type duties)) and personal time (e.g. meal breaks). Oral consent was sought for observation; and formal written consent for qualitative interviews.

### Data analysis

Interviews and workshop proceedings were audio-recorded, transcribed fully, entered onto a database (NVivo 9) for initial coding, sorting and linking. Observations and conversations were captured in contemporaneous jottings, then written up as expanded accounts [22]. Qualitative data were analysed using grounded theory methods: concurrent data collection and analysis, constant comparison, search for disconfirming cases and memo-writing [23, 24]. Ideas about emerging data were tested through discussion among the research team, further data collection and focused analysis. Analysis was recursive moving iteratively between the empirical data, sense making in relation to it and dialogue with the literature, combining reductive (via coding) and connecting (processual/narrative) strategies [25]. Datasets for each ward were combined to create a narrative account of POD implementation planning and delivery in context. Cross-case comparison facilitated an explanatory account of the patterns of variation, including contingencies that impinged on these, and consequences flowing from them, using the conditional matrix as a sensitising framework [24].

Quantitative data were analysed using appropriate parametric and non-parametric statistical methods to give summary descriptions and investigate comparisons. Staff workload data analysis included investigation of the relationship between dependency, activity and other variables [20, 21].

The study was approved by an NHS Research Ethics Committee (10/H1302/66). Before commencing fieldwork, meetings were organised with staff on each participating ward and information leaflets distributed; researchers were also available at designated times to answer questions about the study. Posters about the study were displayed on ward noticeboards and in staff rooms. Participation was voluntary and individuals could withdraw at any time.

## Results

We describe features of organisational contexts within which POD was inserted and examine POD implementation and delivery. We consider whether the theory of change underpinning POD is supported by the findings, critically review feasibility and implications for a future randomised controlled trial to determine clinical outcomes.

Five NHS hospitals in Yorkshire, UK, varying in size and socio-economic context were initially recruited (Table 1).

**Table 1:** Participating hospitals

Hospital	A	B	C	D	E
Number beds	396	c. 900	1113	c. 450	c. 420
Location	Town	City	City	Town	Large market town
Approximate population	76,000 Affluent, predominantly white	500,000 Large ethnic population, areas of high deprivation	750,500 Ethnically diverse; mixed socio-demographic profile	90,000 Formerly manufacture, now in decline; diverse ethnicity	163,000 Urban, ethnically diverse population

### Ward organisational context

*Profile of participating wards:* [see Table 2]*:* Of six wards recruited, ward 5 in Hospital D withdrew prior to commencing preparatory work, citing staffing difficulties. Among the remaining five study wards, three were care of older people and two were surgical orthopaedic. Bed occupancy was high: varying between 89 and 100%. Occupancy rates over 85% have been associated with regular bed shortages, periodic bed crises and increased numbers of hospital-acquired infections [27]; and clinical guidance suggests that bed occupancy exceeding 90% is associated with risk of adverse patient outcomes [28].

**Table 2:** Ward organisational profile

	Hospital A		Hospital A		Hospital B		Hospital C		Hospital E	
Ward	1		2		3		4		6	
Specialty	Care Older People		Surgical orthopaedic		Care Older People		Care Older People		Surgical orthopaedic/ Fracture neck of femur**	
Number of beds	29		23		28		28		31	(22)
Organisation of bed space	3 × 6 bed bays; 1 × 5 bed bay; 6 single rooms		3 × 4 bed bays; 11 single rooms		4 × 4 bed bays; 12 single rooms		4X4 bed bays; 12 single rooms		4 × 6 bed bays; 6 single rooms	2 × 3 bed bays; 3 × 2 rooms; 10 single rooms
Staffing (average day)	1:3.3		1:3.3		1:4.0		1:5.6		1:4.4	1:3.5
Ratio nursing/care day	50:50		50:50		56:44		33:66		57:43	57:43
Staffing (night)	1:7.2		1:7.7		1:7.0		1:9.3		1:6.2	–
Ratio nursing/care night	50:50		66:33		50:50		66:33		40:60	–
Bed occupancy	100%		91%		89%		100%		92%	100%
% moderate/high dependency	55%		52%		80%		89%		60%	90%

* Royal College of Nursing 'safe staffing level on older people wards based on 28 beds [26] is: Skill mix 50:50; RN/patient ratio 1:7; staff/patient ratio 1:3.3-3.8

**The ward changed specialty between the pre-delivery and post- delivery phases

Several wards were understaffed, being most pronounced on care of older people wards, resulting in routine use of bank and agency staff to cover shifts. Based on UK Royal College of Nursing recommendations [26], ward 4 consistently operated below safe care staffing levels during implementation and delivery, and wards 3 and 6 during implementation.

*Patient profile* [see Table 3]*:* On all wards, among those ≥65 years, patients in advanced older age dominated. Patient dependency was high, particularly on care of older people wards (around 90% were dependent on staff for most of their care needs). Lower dependency on trauma orthopaedic wards (around two-thirds had high-dependency needs) reflected a wider age profile on these wards.

**Table 3:** Patient profile by ward

Ward	1		2		3		4		6	
Specialty	Care Older People		Surgical orthopaedic		Care Older People		Care Older People		Surgical orthopaedic/ Fracture neck of femur^a^	
Phase	Pre-delivery	Post-delivery	Pre-delivery	Post-delivery	Pre-delivery	Post-delivery	Pre-delivery	Post-delivery	Pre-delivery	Post-delivery
Period	Jul 11- Dec 11	Jan 12- Jun 12	Jul 11- Dec 11	Jan 12- Jun 12	Jul 11- Apr 12	May 12-Sep 12	Oct 12- May12	Jun 12- Oct12	Sep 12- Dec 12	Jan 13- Apr 13
Duration (months)	6	6	6	6	10	5	8^b^	5	4	4
Number of admissions (aged 65 years and over)	265	245	197	203	724	406	315	321	169	90
Female (%)	188 (71%)	169 (69%)	132 (67%)	147 (72%)	382 (53%)	202 (50%)	315 (100%)	321 (100%)	163 (96%)	77 (86%)
Median (IQR) age	87 (83–90)	86 (82–91)	82 (74–87)	82 (75–87)	84 (80–89)	84 (80–88)	86 (82–90)	87 (83–91)	80 (72–86)	83 (78–88)
Median (IQR) hospital stay	18 (10–32)	17 (10–30)	9 (3–16)	10 (4–23)	8 (4–14)	8 (5–15)	10 (6–20)	10 (6–20)	8 (3–15)	10 (5–21)
Discharge destination										
Usual residence	164 (62%)	141 (58%)	153 (78%)	146 (72%)	427 (59%)	226 (56%)	243 (77%)	235 (73%)	127 (75%)	51 (57%)
Care home	36 (14%)	44 (18%)	26 (13%)	27 (13%)	98 (14%)	54 (13%)	31 (10%)	44 (14%)	18 (11%)	20 (22%)
Other hospital	7 (3%)	4 (2%)	5 (3%)	7 (3%)	74 (10%)	51 (13%)	4 (1%)	1 (0%)	7 (4%)	5 (6%)
Other	22 (8%)	24 (10%)	8 (4%)	16 (8%)	1 (0%)	6 (2%)	3 (1%)	0 (0%)	8 (5%)	6 (7%)
Died	36 (14%)	32 (13%)	5 (3%)	7 (3%)	122 (17%)	69 (17%)	34 (11%)	41 (13%)	9 (5%)	8 (9%)
Missing data	0	0	0	0	2	0	0	0	0	0

^a^The ward changed specialty between the pre-delivery and post- delivery; patient profiles not comparable

^b^Data for pre-delivery only available from Jan 2012 onwards, i.e. 5 months

Although change is ubiquitous in large, complex organisations like the NHS, there were additional sources of turbulence during the research. Most study wards moved physical location or changed their ward model immediately prior to or during POD implementation, as a consequence of local reorganisations.

### Phase 1: planning POD implementation

#### Mobilising human resources

Identifying individuals to lead POD implementation involved discussions with hospital middle managers, practice development leads, clinicians, ward and voluntary services managers. Interest in participation was multiple and varied. Clinicians were keen to increase delirium awareness among nursing and care staff; senior nurses with specialist training in delirium viewed it as opportunity to improve care quality; senior ward staffs’ interest was sparked by volunteer involvement to enhance patients’ hospital experience; and voluntary services staff were enthusiastic about expanding volunteering. Melding these multiple interests toward a common purpose presented an implementation challenge.

#### Leading change

Negotiation centred on identifying at minimum a senior ward team member to facilitate the programme’s reach to ward staff; a voluntary services manager to act as the conduit to volunteer participation; and a middle manager/clinician with positional authority to mobilise human and material resources. Preparatory work in the first site suggested that although the contribution of these stakeholders was necessary to make implementation happen, it was insufficient to drive it; a pro-active site steer was essential. Among ward managers, the exigencies of day-to-day care delivery and managing patient flow constrained their capacity to enact the role; senior clinicians perceived their involvement as maintaining a supportive presence than being active participants; and voluntary services managers viewed their remit as task specific. In hospitals A and E and wards 1, 2 and 6, a matron/deputy matron came forward during early meetings to drive change. Clinical interest in delirium and positional authority shaped their emergence. Capacity for relationship building through vertical and horizontal networking between ward and directorate levels created a facilitative environment, and activated the change process. Whereas the research team initiated meetings, organised workshops and introductory events, it was the backstage work of these ‘drivers’ in negotiating with, and engaging relevant others that proved decisive in pursuing implementation as a collaborative enterprise. They co-ordinated the work of team members in developing, testing and refining systems and paperwork for assessing risk, developing delirium care plans and volunteer roles, and modifying exemplar tools for incorporation into ward/hospital systems. Dedicated time negotiated within their work space made enactment of the driver role feasible.

This form of pro-active leadership proved difficult to achieve on other wards. Attempted in ward 3, it was not carried through. A working group had been established of relevant stakeholders led by a clinical manager, but it was primarily a discussion forum. It was assumed that the ward manager would take charge of implementation. Six months into the preparatory phase, little progress had been made: burdened by nursing staff vacancies, the ward manager engaged intermittently in the group. In ward 4, negotiations about participation were protracted – in a stop/re-start dynamic with periods of resurgent communication between. Staffing difficulties were cited as contributory factors. As a consequence, the research team introduced a modification: financial support to second a staff nurse for the equivalent of one day a week over six months to assist with practical POD preparatory tasks, intended to support but not substitute for, directorate and senior ward staff. Threaded through accounts of POD preparatory work are factors implicated in how this investment worked in particular local contexts and not in others.

#### Preparing for implementation

Implementation planning proceeded as intended in wards 1, 2, 3, and 6, albeit timescales were protracted in wards 3 and 6; it was pursued in ward 4 with some success in some aspects (‘partial implementation’).

##### Reviewing practice: observations/audits

The conduct of observations/audits by implementation teams varied. In wards 1 and 2, they were carried out by different professionals (senior therapists, mental health liaison practitioner, dietician and matron), their respective disciplinary lenses and seniority viewed as enhancing ownership. They were completed by the seconded staff nurse on wards 3 and 4, and on ward 6 by nurses at different levels of seniority and varied experience with older people. Nevertheless, the function observations performed was similar: creating tension for change through ‘seeing’ how practices militated against aspects of quality patient care pertinent to POD delivery:

*It’s been a massive learning curve for me … just in seeing things… there were some patients who didn’t eat because they were waiting so long that the meal was cold when it got to them and they didn’t want it …We were also so busy with the red tray system that patients who might need help but who didn’t have a red tray … could get missed.*

Staff nurse, Ward 4.

Observations highlighted unintended consequences of decisions taken to optimise efficiencies in deploying staff. The following was recorded by the researcher in a field note during a feedback meeting after staff had completed their observations.

Field note, Ward 2.

*The staff member reported that during her observation patients were sat up in bed for dinner and they could reach drinks; tea was poured before the meal arrived and was cold when dinner was served. The ensuing discussion referred to a decision by middle managers to deploy domestic staff in making drinks: because of protected meal times they had to stop cleaning. The meeting concluded that the ward manager would ask domestic staff to help give out meals (something they were not currently doing) and provide a hot drink after the meal.*

Some changes considered by implementation teams as pertinent to delirium prevention, impacted on patient care generally. For example, the pace of work early morning was observed as an extended period of frenetic activity. Ward and visiting staff were described as simultaneously launching into bays and rooms: healthcare assistants to wash and dress patients; nurses to do clinical observations; domestic staff to serve breakfast; doctors to do ward rounds. They then swept out and moved on, leaving patients alone, tired and dozing. Discussed and introduced was an approach to ‘slowing the pace’ by extending the time during which morning work routines were completed, allowing staff time to spend with patients.

Creating tension for change among implementation teams was necessary but insufficient. Contingent on extending POD’s ‘reach’ was that the wider staff team also ‘saw’ the need for change. On wards 1, 2, 3 and 6, via routine and special meetings, ward managers facilitated discussion and reflection on audit findings with their staff. On ward 4, barriers at several levels operated to constrain team engagement: senior staff were preoccupied with managing patient flow with prolonged staffing difficulties, and ‘reach’ was compromised by heavy reliance on bank and agency workers and staff discontinuity.

##### Increasing delirium knowledge

Nursing, therapy and care staff on all wards reported minimal knowledge of delirium and its adverse consequences for patients and caregivers, similar to studies in acute settings elsewhere [29–31]. It was the disruptive behavioural manifestations of hyperactive delirium that were most salient in the problems posed for ward management. The ‘silence’ of hypoactive delirium associated with greater morbidity and mortality [32], contributed to its invisibility among staff. There was little understanding that delirium might be prevented. Exemplar material (leaflets, posters) in the POD manual were designed to draw delirium prevention into the forefront of staff awareness. Creative approaches were adopted: for example, on ward 4, the seconded nurse devised a ‘delirium tree’ in the staff room with branches of ‘notes’ denoting specific POD interventions. Helpful as prompts, these were additional to, but not substitutes for active methods to enhance knowledge and create a facilitative change environment.

On wards 1, 2, 3 and 6, formal training was provided to all staff; the mode of delivery modified to maximise participation. For example, small groups of 3/4 staff with the matron in huddles over several sessions, each of 20 min duration (ward 6); multiple, short 10–15 min ‘breakfast events, led by the seconded nurse until all material was covered with all staff (ward 3); jointly delivered multiple group sessions with a mental health practitioner and senior nurse (wards 1 and 2). Training conveyed delirium knowledge with reflection on staff experience and current practice through the lens of delirium prevention, to engage staff in thinking differently about it.

*We had the meeting to feedback audit observations to staff; but I linked training on delirium with observations and with examples of patients on the floor… We talked about … a patient to whom this was currently happening… and we’d then link the key step on the training programme with the person…Linking knowledge about delirium to experience and practice on the ward…made it very easy to engage staff …in how they could adapt what they do every day to prevent delirium or pick it up early.*

Matron, Hospital E.

A comparison of wards 3 and 4, each with a seconded nurse to steer implementation and lacking pro-active support at directorate level, illustrates the significance of local contingencies in accomplishing planning tasks. The ward 3 nurse had informal support of a senior clinician with authority to negotiate through, and secure resolution of competing priorities that threatened implementation planning. This contrasted with the limited influence the ward 4 nurse could exert on colleagues as a consequence of discontinuity within the staff group and senior ward staff’s lack of capacity to provide support. Absence of training, moreover, meant that delirium prevention was neither understood nor accorded value. Only changes within the nurse’s direct sphere of influence occurred, which included supporting volunteers. Given demand pressures, preventive interventions subject of formal surveillance, accountability and sanctions (e.g. pressure sores and falls) were prioritised.

##### Establishing systems and paperwork

Preparatory work comprised development of systems, processes and associated documentation to identify at-risk patients, establish how staff would act on interventions, communicate with each other, with families and volunteers, and review patients’ progress.

All four full implementer wards incorporated a delirium risk tool into ward admission assessments, conducted by nurses. A care plan, pertaining to identified risks was completed for each patient. POD leads acknowledged that staff participation in new systems and processes required that ‘*we’ve got to see the use of it and what a difference it makes and ideally it’s not going to add to the workload already’.* They prioritised trying out different ways of capturing, recording and reviewing how changes could be adapted to fit local systems for optimal workability. ‘Negotiated experimentation’ (suggesting, eliciting ideas, trialling and reviewing) cultivated staff engagement with system construction, which in turn helped to embed new practices.

##### Enrolling volunteers in POD

Participating hospitals had a long tradition of volunteering, the size of their volunteer base varying between 300 (Hospital E) and 700 (Hospital C). Voluntary services had established systems for recruiting, training and supporting volunteers who performed a diversity of roles in different locations, similar to the national picture [33]: ‘meeting and greeting’ visitors, orienting and responding to requests for information in waiting areas and outpatient departments, providing refreshments and newspapers to in-patients, acting as patient befrienders and helping with mealtimes. There was limited, direct volunteer involvement with older patients at study commencement: three wards had some volunteer ‘befrienders’ and in two of these volunteers provided mealtime assistance to patients.

POD volunteers were not specifically recruited to the programme initially: they were drawn from among those who had applied to become volunteers and had expressed interest in working with older people. Motivations were diverse: students wishing to pursue a career as a health professional; working age young people and adults to enhance their employability; retired people seeking something meaningful to do; and those who wished to support the hospital for the care they or a relative had received.

##### POD volunteer training and role assignment

Involving volunteers in POD encompassed reciprocal agreement between ward staff and voluntary services on work roles, competencies and training to enact them; establishing methods for communicating information about which patients to work with, in relation to what tasks; creating mutually supportive working arrangements, and engendering a sense of common purpose.

Volunteer specific training was aimed at reinforcing POD as a collaboration between staff and volunteers. This conception, however, was not carried through in all sites. POD specific training was pursued on wards 1 and 2 only. On these wards, additional to addressing hospital procedural rules, typically on infection control, confidentiality and patient safety, training articulated the purpose and content of the POD system of care, placed emphasis on staff and volunteers working together on a common objective, and contextualised reciprocal volunteer/staff communication and staff support within that joint endeavour.

On wards 3, 4 and 6, standard hospital volunteer training was supplemented with an information leaflet about POD, although ward 6 later altered its approach. On wards 4 and 6, training was limited to hospital procedural rules. On ward 3, it was more expansive: task specific training depending on role, communication skills and dementia awareness. Their rationale for not deviating from standard training was pragmatic: they nether had the resources to do things differently nor did they see the need for it. Their existing model facilitated substitutability of volunteers across the diversity of roles performed in a hospital context: if the person did not ‘fit’ in one service environment, s/he might ‘fit’ in another. Given the investment of voluntary services staff in recruiting and training volunteers, substitutability was viewed as important.

*Even if the volunteer doesn’t feel they are in the right environment…they can fit into a different type of voluntary work. It doesn’t mean we lose them.*

Voluntary service manager, Hospital C.

On these wards, voluntary services established from the outset the tasks volunteers would perform and for which training prepared them. Although not dissimilar to POD role descriptions, they were conveyed as incidental to, rather than central to delirium prevention.

*They follow a task description that … we use... And I didn’t want to detract them from that, I wanted them to carry that on.*

Voluntary services manager, Hospital B.

On wards 1 and 2, volunteer training was followed by informal introductory meetings with ward staff in a social setting. On ward 4, voluntary services organised a similar gathering to introduce volunteers to staff and to familiarise them with the ward environment. On wards 3 and 6, usual practice pertained: a voluntary services staff member accompanied the volunteer the first occasion s/he went on to the ward.

At conclusion of the preparatory phase, all five wards had recruited varied numbers of volunteers: 18 for wards 1 and 2, nine and ten respectively for wards 3 and 4 and five for ward 6, where recruitment was ongoing. Core features of the role were: providing emotional and social support, engaging in stimulating activities, ensuring patients were drinking enough and providing practical assistance as needed. On wards 1 and 2, therapists developed guidance for volunteers to assist patients with what they termed ‘social mobility’, accompanying those able to walk independently but lacking confidence to do so.

It was intended that volunteers would work one 2/3 h session weekly. Assigning volunteers to patients and tasks was to occur via the patient care plan, and a symbol on the patient board identifying delirium. Volunteers were to complete the care plan at each visit; wards also maintained a book for them to raise issues/make comments.

### Phase 2: delivering POD

POD was fully delivered in wards 1, 2, 3 and 6 over six months. Delivery involved some adaptation of the systems and processes established during the preparatory phase as new challenges unfolded requiring resolution.

#### Change in delirium knowledge and practice

On these four POD wards, enhanced knowledge of delirium had seeped into work practices prior to delivery through knowledge sharing and practice reflection. Increasing use of the term ‘delirium’ instead of ‘confusion’ was symbolic of new understanding with consequences for action.

*Slowly you started noticing at handovers …that staff… wouldn’t say Mrs X was confused; the word ‘confused’ went, and people talked about whether she may have a delirium…it became a clinical thing, not something to be dismissed…delirium wasn’t just about the person walking up and down…they were picking up on people who were quiet. They started associating a patient’s sudden change in behaviour with possible delirium and doing something about it.*

Senior nurse, Ward 6.

Staff suggested that delirium knowledge facilitated empathic connection with the experience of patients as opposed to ‘*just seeing a ‘problem’ patient*’. Whereas nursing and care staff might have previously reacted impatiently to a patient *“behaving badly,”* they were more likely to spend time with the patient, seeking to ease their confusion and distress.

The new systems and paperwork aligned with delirium knowledge projected a collective sense of thinking and acting on delirium prevention. Since most patients on care of older people wards and older patients on trauma orthopaedic wards met criteria for delirium risk, the risk tool’s primary function was to reflexively produce an individual patient care plan specifying action to be taken on each risk factor identified:

*It was a means to pull staff through the nursing process: you’ve assessed someone’s risk, now it’s about planning work relating to it, evaluating what you’ve done and what’s happened as a result… the difference between putting a tick beside hygiene care and not thinking what it means.*

Matron, Hospital E.

Work relating to delirium care plans served to extend action in respect of other aspects of routine practice. For example, patients at risk of malnutrition were identified through the Malnutrition Universal Screening Tool (MUST) on ward admission, triggering a dietician assessment. For nurses doing the admission assessment, identifying a patient at risk of poor nutrition in the delirium care plan meant that action on nutrition encompassed those who might require encouragement or assistance to eat.

Care plans were reviewed regularly by senior ward nurses. Compliance was fostered through review of action during daily ‘handovers’ with nursing and care staff. Changes in behaviour patterns were noted and implications for further action considered. The division of labour on delirium prevention highlighted the contribution of health care assistants because of their direct patient knowledge. This in turn enhanced confidence in their ability to note, make sense of, and draw attention of qualified staff to subtle changes in patients that required concerted action.

*Our ‘Health Cares’ are coming to us nurses and saying, ‘look this patient has only taken sips of fluid, we need to work on this together’*… *we were always notoriously bad at filling in fluid balance charts …but now hydration, glasses have become more part of the general routine…there’s a purpose to it.*

Staff nurse, Ward 3.

Attaching meaning actions by locating them in context of delirium prevention was important in reinforcing practice change.

Direct ward engagement in POD delivery did not extend to therapists, except for ward 6, where a senior therapist was in the implementation team. It was planned that cognitive stimulation for patients with dementia, at particularly high risk of developing delirium, would be prioritised. This was enacted through deploying student occupational therapists (OTs) in a different way. Instead of focusing on what had become their core activity: ‘*discharges, equipment, home visits’,* OT students worked therapeutically with cognitively impaired patients at risk of delirium. Transforming the day room into a therapeutic space, OTs used reminiscence materials, games, music, and conversation to connect patients with their pre-hospital lives and relationships. They involved relatives directly in eliciting knowledge about patients’ interests and preferences, encouraging them to bring in favourite music, videos and books. The organisational context in part facilitated this focus. As an orthopaedic trauma ward, dedicated therapists with a visible ongoing presence were the norm, contrasting with care of older people wards where therapists were not physically located there, but attending to work with individual patients and for multidisciplinary meetings.

On ward 4, although improvements in some aspects of care had occurred as a consequence of preparatory work (for example, more timely delivery of meals), systematic engagement of the ward team in delirium prevention did not happen, reinforcing the significance of implementation work in creating a facilitative change environment, embracing the wider staff team.

#### Integrating volunteers

Preparatory work had generated considerable enthusiasm for volunteers among staff who had invested time in creating a welcoming and supportive environment, to ensure the smooth integration of volunteers onto the ward. In practice effecting integration was more complicated.

The first weeks of delivery were especially vulnerable to volunteer attrition. Between a fifth and a third of volunteers never started or left after a few sessions either for personal reasons (illness, pressure of work) or because they found working with older people too difficult. Personal experience in caring for older people could be a hindrance or a spur to sustaining volunteering: the former where it unearthed painful emotions; the latter when it generated confidence in care tasks and empathic connection with the patient as person.

Even those who persisted, found it intimidating in the beginning.

*Going onto a ward where there are quite a lot of really ill people …I’d done loads of preparation for it and I still found it daunting so, yeah. But I think once you’ve done it once or twice you kind of know what it’s like and you know what to do…and staff were welcoming… helpful and reassuring and showed me what to do …until they knew I’d got the hang of it.*

Student volunteer 01, Ward 1.

Unfamiliarity of the environment and in engaging with older patients, particularly those who were frail or had a cognitive impairment, provoked anxiety which took knowledge and experience to overcome.

*It was difficult …with a patient with dementia I didn’t know how to handle the situation…more often because of their distress…I learned how to get into a conversation… it can seem disjointed…but you learn to listen to the flow and relate to that…there is a massive sense of fulfilment.*

Student volunteer 03, Ward 6.

Volunteers with expertise in caring for older people either by personal experience or previous employment, found that negotiating the boundary between ‘being helpful’ and not undermining staff, was tricky:

*It’s taking me a long time to get to know the staff because they’re busy, they turnover… I can ask them but if they’ve got jobs to do … It’s difficult isn’t it …I have to be a little bit careful that I’m not treading on the toes of the healthcare assistants and I’m conscious of that and … occasionally I feel by being eager I can be seen to chase their tails when they’re feeling very busy and because I’m only in for a short time – about 6 h – I can afford to be walking around and available anytime I’m on the ward but it’s hardly sustainable for the staff to do that …but if I see someone in a pickle I want to go and help...*

Retired volunteer 04, Ward 4.

Nevertheless, there were multiple examples of how volunteers developed creative and person-centred approaches to communicate with frail and cognitively impaired older people not as patients but as individuals with a past, present and fears and hopes for a future. They supported at-risk patients in eating meals together in a social space that also involved assisting them to move beyond the bed/bay. In several wards, volunteers worked closely with individual staff: on ward 2, the creation of a communal dining space for patients was initiated jointly with the nutrition assistant, who had assumed day-to-day responsibility for supporting volunteers. On ward 6, volunteers worked alongside OTs in group and individual reminiscence and social activities. Among volunteers in wards 1, 2 and 6, there was a strong sense of involvement in a joint programme with staff. In ward 4 by contrast, high level commitment of several retired volunteers was animated by their personal values of affording dignity in older age.

The number of hours contributed weekly to each ward by volunteers was modest (mean 8 h, range 4 to 15), although this is likely an underestimate (time spent by those leaving after a short time was not always recorded). This did not reflect lack of individual commitment: some contributed up to eight hours a week. On ward 3, where few volunteers took part, two completed ≈56 of the 81 h recorded during delivery; at the other end of the spectrum one volunteer did not return after the first shift, and the second left after completing two shifts.

Except for ward 6, voluntary services did not pursue recruitment after POD preparatory implementation. Wards tried out different methods to support and retain volunteers with mixed success: ‘buddying’ a new volunteer with one who was experienced; organising feedback meetings to identify need for support. In absence of ongoing recruitment and with attrition on these wards, under a third of the original cohort remained to end of delivery, of whom most were planning to continue.

On ward 6, from an inauspicious start (only two of five volunteers started and one left shortly after to take up employment), volunteer numbers slowly built up by adopting novel recruitment methods and establishing bespoke training and support structures. A direct approach to a local 6th form college, led to recruitment of first one, then three further students interested in a health career. Each new volunteer was taken under the wing of a senior staff member and spent the first couple of weeks shadowing ward staff and an ‘experienced’ volunteer until confident of managing independently. Reciprocal peer support was fostered; encouraging a ‘link’ experienced volunteer to assume responsibility for it, made it happen. Recruitment and training were re-worked toward the end of delivery for a new volunteer intake: one-to-one interview with a senior nurse; then a dedicated, half-day POD training event with ward senior staff and older person’s lead. Within six months of the delivery phase ending, a further eight student volunteers, additional to the four who continued beyond the study end, were working on the ward. Persistence of staff in investing in new methods for recruiting, training and supporting volunteers was underpinned by commitment to sustaining POD and extending it to other wards.

#### Workability of POD delivery

Structured ward observations demonstrated only small changes in nursing input in direct patient care (Additional file 1 Staff workload) with a 4% increase for ward sisters and staff nurses and a 2% decrease for support workers (partly due to the absence of a ward clerk during delivery on one ward and care staff assuming aspects of the role). There were small decreases in the percentage of ‘associated work’ (15 to 13%) and ‘personal time‘(13 to 12%) undertaken by nursing staff.

#### Delivery as a non-linear process

The process of practice change during delivery was non-linear and subject to qualitative leaps forward and steps backward. It required ongoing appraisal and testing out solutions to emerging problems, as ward 6 exemplified. Local contextual factors impinging directly on wards could also disrupt delivery. Less than two months into delivery, Hospital A initiated a consultation on reducing care of older people beds. This involved reduction of half the beds on ward 1 and the creation of a mixed ward in a different location, with a similar number of patients from an adjacent, specialist medical ward. Strong representations from clinical and nursing staff resulted in senior managers delaying re-organisation until after POD delivery. Nevertheless, by month 4, despite the depth and reach of preparatory work, staff struggled to sustain POD: bed closures had begun with adverse impact on staff morale; the requirement on all staff to re-apply for posts in the new structure created uncertainty; the ward manager was preoccupied with organising the transition; and prolonged norovirus infections resulting in temporary bay closures disrupted continuity of volunteer visits. This was additional to the loss of patients to work with as bays closed anticipatory of the ward move. Staff described this period as a ‘*nightmare’.* The remaining three full-delivery wards, continued with POD post-delivery.

## Discussion

This study examined the feasibility of implementation planning and delivery of a novel, theory informed, multicomponent delirium prevention system of care (POD) in NHS hospital wards, an exemplar of a complex intervention [34]. Although delirium preventive interventions comprise practices that pertain to features of quality, person-centred care, purposive and systematic engagement in such practices are situationally dependent on delirium knowledge, skills and resources.

The preparatory work of POD implementation was fully accomplished in four of five participating wards, and subsequently delivered over six months in those wards. The implementation strategy components worked synergistically, interactively and broadly as intended to effect the hypothesised mechanisms of change theorised in NPT, albeit in specific conditions. Thus, observations of practice pertinent to each of the preventive interventions using the audit tool served the function of enabling staff to ‘see’ how and in what ways intervention delivery was similar to and different from existing practice. Creating tension for practice change only occurred in circumstances where what was observed and the implications for action were conveyed and reviewed within the wider staff team. Developing understanding of delirium through education and training was more than conveying knowledge of its presentation and risk factors. It required engagement of staff in thinking differently about it. Knowledge aligned with interpreting experience against a new mental model facilitated organisational learning and forging networks of practice, as elsewhere [35]. Re-visioning the ‘problem’ of delirium in terms of the consequential distress on patients and the possibility of reducing incident delirium gave meaning and purpose to preventive action through routine care work. Establishing systems and processes for integrating delirium prevention work as an individual and collective accomplishment supported cultural and practice change; and employing ‘negotiated experimentation’ to involve ward staff directly in creating, appraising and modifying systems and practices, enhanced ownership and commitment. It also served to allay fears that it would result in yet more paperwork. We found evidence that this engagement and flexibility fostered creativity and a problem-solving approach.

A major study learning point was that activating the mechanisms of action to implement change required a particular form of leadership to drive it: individuals with formal authority from their organisational position to enlist stakeholders in the work; skills in networking, negotiating and engaging relevant others; and headspace to enact the role. Alternatively, the introduction of an experienced staff nurse with limited dedicated time could organise and accomplish planning tasks in the absence of such a steer in specific conditions: assistance of a senior professional or middle manager to unblock barriers arising from competing hospital priorities; and support from senior ward staff to facilitate engagement of the wider staff group. In ward 4, where the resource of a dedicated nurse to pursue change had marginal ward-level impact, the absence of local leadership and staffing difficulties created an environment in which staff had neither the support nor the headspace to pursue preparatory POD implementation.

Subsequently, delivering POD shifted responsibility to senior ward staff as the primary drivers. The transition from implementation planning to delivery was viewed in hindsight as requiring focused work: to ensure that the systemic, relational and practice dimensions of delirium prevention were being pursued and that the systems and processes in place to embed POD in organisational routines were operating to secure intended benefit. This highlights ongoing appraisal work as critical to facilitating and maintaining cultural and practice change [15, 36]. Moreover, although the distinction between preparation and delivery phases is useful as a heuristic to define types of work and forms of leadership at different time points, delivery is not a simple, fixed accomplishment of the preparatory work but interfaces with local context in unpredictable ways. As others have argued [37–40] implementation of a complex intervention is most appropriately conceived of as an event in a multi-layered and dynamic, complex system which is part of the programme of change and interacts with it to facilitate, mediate or subvert it. This directs attention on how the intervention interfaces with its local context, the dynamic nature of which reinforces the idea of implementation as a process carried forward in and through time, place and setting.

In this study, three broad contextual features were critical in the work of preparing for, and delivering POD: structure, organisation and timing. Structural features of ward contexts, especially staffing difficulties, impeded preparatory implementation and POD delivery, even in the presence of interest and enthusiasm among senior staff to make it happen. Thus, ward 4 without a basic level of staffing (and staff continuity) was unable to engage with the work involved. Adverse organisational factors, such as ward re-organisation, change in location and discontinuity among senior ward staff risked subverting POD implementation and delivery (ward 6). Here, although preparatory POD implementation work was extended in time, the configuration of human resources mobilised in this site cut through organisational constraints: senior management investment to procure dedicated time of a matron to steer implementation; support throughout from the directorate lead for older people and a new senior ward team who embraced the intervention as an opportunity to enhance practice. ‘Timing’ was another contextual factor interfacing with POD which could either facilitate or subvert implementation and delivery. The proposed closure of ward 1, raised in the first weeks of delivery, preoccupied staff and adversely affected morale at a critical point in the change process. Although closure was postponed, structural and organisational factors (bed closures and staff moves) resulted in POD gradually fading away. It is possible that if the proposed changes had occurred later on when POD was more integrated into ward routines, the move to the new ward might have temporarily disrupted POD but not effaced it.

The mechanisms of change to implement and deliver POD demonstrated potential to change ward practice with positive impact on staff, staff/patient interaction and practices pertaining to delirium prevention. This was achieved with modest resources to support preparatory implementation work and transition to delivery (equivalent of one day a week of a ‘driver’).

Our model of delirium prevention included a role for hospital volunteers to enhance ward practice in delirium prevention, particularly regarding those tasks that appear difficult for staff to undertake consistently (e.g. orientation, sustained conversation, meaningful activities). Although volunteer involvement was highly valued by staff, recruitment and retaining volunteers was more difficult than anticipated. Wards varied in the numbers recruited at outset and volunteer attrition was high, with specific points of vulnerability: between recruitment and starting; shortly after starting; then a slower, less predictable drift. Different factors appeared to operate at each point: where general interest collided with the reality of the commitment involved; where engagement with older people in an acute care context was found to be too difficult; and changing circumstances (illness, taking up paid employment, moving away). For some, their motivation to become volunteers could be both a barrier to persistence and a source of sustainability. In three of the four full delivery wards, between a quarter and a third of those indicating interest in volunteering persisted to the six months end, and most intended to continue. The time contribution of individual volunteers was enormously varied. This was the case in wards in which implementation teams had invested heavily in processes and action to establish a supportive environment for volunteers and to involve them conjointly with staff in delivering POD; and in wards in which an integrated approach, for various reasons, did not happen. Different methods introduced to support volunteers had positive effects in sustaining involvement in some contexts but not in others. The pattern of attrition was not dissimilar to that in a recent study of mealtime assistance [41].

There is considerable descriptive knowledge about the motivations and characteristics of volunteers and the benefits they derive from, and contribute to individuals and organisations, through volunteering, from North American [42, 43] and UK research [33, 44]. Within NHS hospitals, involvement of volunteers is uneven and diverse in scope and roles [33, 44]. Direct, purposive support to frail older patients, the majority of in-patients, is a small and variable component of the total. There is a dearth of research on sustainability or benefit for these patients from systematic reviews on provision of assistance with single tasks (mealtime assistance, mobilisation) [45, 46] or providing general support to patients with frailty [47]. There is poor understanding of the dynamic of volunteering in specific contexts: how people start, continue or stop volunteering, what shapes individuals’ volunteer career and what kind of infrastructure is required to co-ordinate and support volunteering in health settings.

### Limitations

Our study primarily addressed the work of preparing implementation and enacting delivery through the lens of those involved. Although we interviewed volunteers about their experience with the programme, this only included those who persisted to end of delivery. Similarly, interviews with ward staff over time would have provided insight into the processes whereby practice change occurred from their different perspectives. Nevertheless, strengths of the study included informal observation of preparatory implementation activities and work practices over time, structured observation of work and workload at different time points and completion of documentation pertaining to new systems and processes.

## Conclusions

We conclude that POD is feasible to implement and deliver in an NHS ward context in specific conditions which we have encapsulated as ‘readiness factors’:

### Leadership in the form of a ‘driver’ and ‘facilitators’

A named, individual ‘driver’ at senior level whose professional authority and vertical networks legitimate the work of POD implementation in face of competing priorities. A ward-based ‘facilitator’, typically the ward manager, to provide support and encouragement to legitimate staff time devoted to POD and extend its reach to the wider staff team. A voluntary services manager to recruit and support volunteers and facilitate their introduction to the ward and POD.

### Dedicated time of an experienced staff member

Dedicated time of around one day per week for around four months to support the day-to-day tasks of implementation and transitioning to delivery.

### Adequate staffing levels

Implementation of what is an augmented system of care requires capacity and resources to deliver at least a basic standard of care. Staffing difficulties including reliance on bank or agency staff means that there is not the headspace for staff to pursue cultural and practice change.

These criteria do not imply knowledge, interest, or prior work on delirium prevention. Rather, they relate to the presence of contingent factors that are necessary to allow selection of wards which are realistically going to be able to implement this complex intervention.

Our findings resulted in a re-assessment of the role of volunteers within delirium prevention in the feasibility trial. The model of delirium prevention we had adopted and described here included a prominent role for hospital volunteers. As most wards were unable to recruit or sustain the number of volunteers needed to have a major impact on delivery; the revised model adopted a flexible approach to inclusion of volunteers.

## Supplementary information

**Additional file 1.** Staff Workload.

## Data Availability

The data that support the findings of this study are available from the corresponding author upon a reasonable request but restrictions apply to the availability of these data on account of ethical requirements regarding anonymity, and so are not publicly available.

## Abbreviations

NHS: National health service
NICE: National institute of health and care excellence
NPT: Normalization process theory
POD: Prevention of delirium system of care
