# Supplementary material for: Process of implementing and delivering the Prevention of Delirium system of care: a mixed method preliminary study
Source: BMC Geriatr. 2019 Dec 31;20:1. doi: 10.1186/s12877-019-1374-x (PMC6938603; doi:10.1186/s12877-019-1374-x)
Supplement: Supplementary file 1 — Additional file 1. Staff Workload. [file 12877_2019_1374_MOESM1_ESM.docx]

**SUPPLEMENTARY APPENDIX : STAFF WORKLOAD**

**Impact of the intervention on nurse workload**

We obtained ward nurse workload data at the start of the POD implementation phase and during the delivery phase of POD implementation on participating wards to gauge the impact of POD on ward staff activity and modification of workload. We used the ‘dependency-acuity’ method, a standardised approach previously developed by a co-applicant and widely used within the NHS (seventy-eight trusts; 2,800 wards, departments and teams). The approach is based on previous workload planning techniques but has the additional advantage of taking into account the dependency and acuity of patients in calculating the workload burden. This approach combines ward-based observations of the activities of staff, undertaken by non-participant observers, linked to the dependency/acuity of patients to produce an overall assessment of the ward staff activity. To obtain a broad sample of nurses’ workload, we undertook ward observations during the 24 hour period. We undertook the ward observations during six shifts (two early; two late; and two night shifts. These observations were undertaken during the implementation phase, i.e. before POD had become established on the ward and during the delivery phase when POD had been in use on the wards for some time.

Data collection consisted of:

**a. A record of ward bed occupancy**

**b. A measure of the dependency/acuity of patients on nursing staff to meet their needs.**

As the dependency/acuity of patients affects the workload burden of staff it was important to take account of this within the analysis. The method we used utilised a simple indicator to signify patients’ reliance on nurses to meet their needs. Patient were rated, in consultation with a senior member of the ward nursing team,on each of the categories in the table below on a score of 1 to 4 (lower to higher dependency):

| **Table.** **Dependency Assessment** | |
| --- | --- |
|  | **Score** |
|  |  |
| **Nursing Attention** |  |
| a) Constant | 4 |
| b) Two hourly or more | 3 |
| c) Four hourly | 2 |
| d) Twice daily or less | 1 |
| **Washing and Dressing** |  |
| a) Daily bed bath or open bath needing two carers | 4 |
| b) Daily bath needing one carer | 3 |
| c) Assistance needed to wash and dress | 2 |
| d) Independent - relative attends to needs | 1 |
| **Using the Toilet** |  |
| a) Incontinent or catheterised | 4 |
| b) Four hourly or more help needed to use the toilet | 3 |
| c) Needs help to use the toilet | 2 |
| d) Independent | 1 |
| **Moving** |  |
| a) Immobile | 4 |
| b) Two carers needed to help patient walk or move around | 3 |
| c) Needs help to walk or move around | 2 |
| d) Independent | 1 |
| **Eating and Drinking** |  |
| a) Fed artificially (e.g., naso-gastrically, intravenously) | 4 |
| b) Depends totally on carer to eat and drink | 3 |
| c) Needs help to eat and drink | 2 |
| d) Independent once meal is served | 1 |
| **Pressure Area Care** |  |
| a) Necrotic areas | 4 |
| b) High risk and needing two hourly or more care | 3 |
| c) Moderate risk needing four hourly care | 2 |
| d) Low risk needing twice-daily check or less | 1 |
| **Relatives** |  |
| a) Relative needs constant explanation/reassurance/support/help | 4 |
| b) Relative needs frequent help/support | 3 |
| c) Relative needs occasional help/support | 2 |
| d) Minimum help/support needed | 1 |

Scores were summated to produce an overall level of dependency:

1. Independent (6-7)

2. Between independent and dependent (8-13)

3. Dependent (14-22)

4. Highly dependent (23-24)

Patients with a dependency/acuity level of 1 were virtually independent of nurses. Patients with a dependency level of 4, on the other hand, were dependent on nurses for most if not all of their needs.

**c. Recording of ward nursing staff activity**

The activity and grade of each nursing member of staff and each volunteer was recorded by the non-participant observer at ten minute intervals. Activites were chosen from a pre-determined list under the four headings: direct face-to-face care; indirect care; associated work; non-productive time. Direct face-to-face care comprised 15 activities; indirect care comprised five activities ; associated work comprised eight activities ; and non-productive time comprised four activities: personal, unoccupied,

Breaks, other.

| **Table. Staff activities and definitions** | |
| --- | --- |
| **Activity** | **Definition** |
| ***Direct Care*** |  |
| Outpatient | Care of an outpatient on ward. |
| Medical Procedures | Extended-role procedure. |
| Communicating with a patient | Including support/ teaching/showing/explaining/assessing/observing. |
| Nutrition | Help with diet and fluids including via NG/ PEG tubes, and including supplements/ special diets. |
| Hygiene | Assist with hygiene and comfort cares and preventative pressure area care. |
| Elimination | Assist/assess/record all excreted fluids/matter. |
| Medication | Administrate by all routes. Check, record, monitor, maintain equipment. Monitor self-medication. |
| Movement | Assist in/around bed and ward, including transferring and performance of exercises. |
| Vital Signs | Measure, monitor, record and interpret TPR/ BP/sats/blood sugar/neurological signs, weight |
| Specimens | Obtain specimens for laboratory/ward testing. |
| Nursing procedure | Hand wash before after patient care. Prepare equipment for treatments. Perform nursing procedures eg. dressings, catheterisation, enemas, pressure area care. Ensure treatments applied and maintained. Care of the deceased and their families. |
| Escorting/ Admitting/ Discharging | Assist in safe transfer/discharge including plan, check-identity and complete documentation. Admit or discharge to/from the ward. Discharge planning. Transfer a body to the mortuary |
| Teaching | Instruct patients. |
| Assisting doctors | On ward round and during procedures/care. |
| Assisting others | With patient intervention/treatments. |
|  |  |
| ***Indirect care*** |  |
| Charting | Commence/ maintain nursing records. |
| Reporting | Give/receive patient information (handovers, MDT. Use of computer PAS system for recording /retrieving patient information. |
| Communicating with staff | Liaison with other health/social care professionals re specific patient requirements. |
| Communicating with relatives | Support and information regarding a patient and any other issues. |
| Teaching | Receive or provide professional/work related instruction or assessment |
|  |  |
| ***Associated activities*** |  |
| Cleaning | Organise, tidy, clean ward areas not associated with specific patient care. Empty bins and sharps bins. Dispose of soiled linen. Clean equipment and furnishings. |
| Meals and drinks | Prepare for and participate in meals and drinks distribution/clearing. Change water jugs. |
| Clerical | Menu lists, patient dependency records, daily bed returns, other clerical work including notes and identification bracelets etc. Use of computer, not patient details/info. |
| Communication | Administrating paperwork and phone calls (including advice line calls) |
| Errands off ward | Deliver/collect/look for items/person etc. |
| Supplies | Safety checks on equipment. Maintain ward supplies, restock emergency trolleys etc. |
| Meeting/In-service training | Attend management and administrative meetings. |
| Supervision/Mentoring | Supervise staff, complete staff reports and appraisals. Orientate new staff members. |
|  |  |
| ***Non-productive time*** |  |
| Personal |  |
| Unoccupied |  |
| Breaks |  |
| Other |  |

Data collection was undertaken by three members of the research team (two nurses and one physiotherapist) who had previously been instructed in the method by the developer of the approach and had undertaken practice sessions in its use.

Analysis

Data was inputted on to an excel spreadsheet and analysed to generate the following:

1. Bed occupancy

This was a measure of how full the ward was, an important consideration when comparing results across time.

1. Patient dependency/acuity

The dependency of patients affects the workload of staff. It was therefore important that this was taken into account within the analysis. We used a simple indicator to assess patients’ reliance on nurses to meet their needs described above.

1. Workload index

The workload index is a single value calculated from bed occupancy, patient dependency and direct care time. It indicates how ‘busy’ the ward care team is. Higher workload values indicate busier wards (in terms of workload).

Dependency data and staff activity data were entered onto an excel spreadsheet. Analysis was undertaken by the developer of the approach (KH) to compare nurse workload before and after POD implementation for all wards and for each ward separately.

**RESULTS**

**Table Supp.1. Staff and volunteer observations and activity by category**

|  | Staff grade | | | | | | Volunteer | | Total | |
| --- | --- | --- | --- | --- | --- | --- | --- | --- | --- | --- |
|  | Manager | | Staff nurse | | Support staff | |  | |  | |
| Phase^a^: | 1 | 2 | 1 | 2 | 1 | 2 | 1 | 2 | 1^b^ | 2 ^b^ |
| Observations: |  |  |  |  |  |  |  |  |  |  |
| N | 485 | 392 | 3338 | 2762 | 3633 | 2804 | 28 | 88 | 8257 | 6711 |
| % | 6 | 6 | 45 | 46 | 49 | 46 | 0 | 1 | 100 | 100 |
| Activity category: |  |  |  |  |  |  |  |  |  |  |
| Direct care (%) | 21 | 25 | 42 | 46 | 48 | 46 | 82 | 63 | 45 | 46 |
| Indirect care (%) | 46 | 45 | 38 | 36 | 18 | 21 | 0 | 13 | 28 | 29 |
| Associat care (%) | 27 | 23 | 9 | 7 | 19 | 20 | 18 | 14 | 15 | 13 |
| Personal time (%) | 5 | 7 | 11 | 11 | 16 | 14 | 0 | 11 | 13 | 12 |
| ^a^1=pre-POD; 2=post-POD; ^b^ Totals 1 and 2 include supernummary students, which do not have a column in the staff grade data. | | | | | | | | | | |
